# Exosomal HBV-DNA for diagnosis and treatment monitoring of chronic hepatitis B

**DOI:** 10.1515/biol-2022-0585

**Published:** 2023-04-15

**Authors:** Xu Xu, Li Zhang, Jiamin Liu, Xiangxin Kong, Yu Yin, Zhiwei Jia, Xiaoqin Zhang, Bin Peng, Min Ji, Wanlong Pan

**Affiliations:** Experimental Teaching Center for Pathogen Biology and Immunology & Department of Microbiology and Immunology, North Sichuan Medical College, Nanchong, Sichuan, 637100, China; Emergency Department, Affiliated Hospital of North Sichuan Medical College, Nanchong, Sichuan, 637000, China; Department of Intensive Care Medicine, Affiliated Hospital of North Sichuan Medical College, Sichuan, 637000, China; School of Basic Medicine, North Sichuan Medical College, Nanchong, Sichuan, 637100, China; People’s Hospital of Jianyang, Chengdu, Sichuan, 641400, China

**Keywords:** exosomes, serum, hepatitis B, virus, DNA

## Abstract

This study examined exosomal hepatitis B virus (HBV)-DNA levels in chronic HBV infection (CHB). Patients were grouped according to the European Association for the Study of the Liver classification (1: HBV-DNA-positive CHB, normal alanine aminotransferase [ALT]; 2: HBV-DNA-positive CHB, elevated ALT; 3: HBV-DNA-negative HBeAb-positive CHB, normal ALT; 4: HBV-DNA-positive HBeAg-negative HBeAb-positive CHB, elevated ALT; 5: HBV-DNA-negative, HBcAb-positive; 6: HBV-negative, normal ALT). Exosomes were isolated, comparative analysis of exosomes and serum HBV-DNA. The HBV-DNA content was lower in exosomes than in serum for groups 1, 2, and 4 (all *P* < 0.05). In the groups negative for serum HBV-DNA (groups 3 and 5), the exosomal HBV-DNA levels were higher than the serum HBV-DNA levels (all *P* < 0.05). The exosomal and serum HBV-DNA levels were correlated in groups 2 (*R* ^2^ = 0.84) and 4 (*R* ^2^ = 0.98). The exosomal HBV-DNA levels were correlated with total bilirubin (*R* ^2^ = 0.94), direct bilirubin (*R* ^2^ = 0.82), and indirect bilirubin (*R* ^2^ = 0.81) in group 5 (all *P* < 0.05). In patients with CHB and negative for serum HBV-DNA, exosomal HBV-DNA was detectable and could be used to monitor the treatment effects. Exosomal HBV-DNA could be used in patients with a high suspicion of HBV infection but negative for serum HBV-DNA.

## 1 Introduction

About 292 million people are infected with the hepatitis B virus (HBV) in the world, with the highest infection rates being observed in Africa and the Western Pacific region, where the virus-carrying rates are >6% [1]. China is a hard-hit country, and HBV carriers account for about 6% of the total Chinese population [1].

Presently, diagnosing hepatitis B is mainly based on the HBV-DNA nucleic acid test, HBV serum marker test, liver function test, and other auxiliary tests [2]. The latest version of the European HBV treatment guidelines [3] stipulates that the treatment criteria are met only when both HBV-DNA and liver function indicators (like alanine aminotransferase [ALT]) are elevated. The detection of low levels of HBV-DNA replication depends on liver biopsy [2,3], which is invasive, difficult for the patients to accept and cannot be used repeatedly for treatment effect monitoring.

Exosomes are small (40–200 nm) vesicles containing complex RNA, DNA, and proteins and are formed by the fusion and exocytosis of intracellular vesicles and plasma membrane through multiple vesicle bodies [4,5,6], first detected in humans by Caby et al. [7]. Exosomes mainly promote cell interactions through receptor-mediated endocytosis by the target cells [8,9]. Exosomes cannot be degraded by the enzymes found in body fluids due to the structure of their bimolecular lipid layer [10]. Therefore, the proteins, viruses, nucleic acids, and other substances carried by exosomes will not be found in the body fluids [10]. These features contribute to easy isolation and detection at all stages or even early stages of the disease [11,12,13]. Therefore, exosomes can be a medium for virus replication, transmission, and regulation [9,14]. Exosomes are potentially more sensitive indicators than serum markers to reflect the occurrence, development, and outcome of diseases and might provide treatment reference indicators with more clinical significance [9,14].

Based on this assumption, this study aimed to examine the sensitivity of exosomal HBV-DNA levels and their correlation with serum biochemical indicators of HBV infection.

## 2 Materials and methods

### 2.1 Patients and samples

Serum HBV-DNA (HBV-DNA nucleic acid detection kit, Shanghai Kehua), immune indicators (HBsAg, HBsAb, HBeAg, HBeAb, and HBcAb, Hepatitis B surface antigen Assay Detection Kit, Roche), and liver biochemical indicators (total bilirubin [TBIL], direct bilirubin [DBIL], indirect bilirubin [IBIL], and ALT) were determined in the serum samples of 39 patients at the Affiliated Hospital of North Sichuan Medical College. The study involved human participants and was reviewed and approved by North Sichuan Medical College-2020 NSMC, number 10. Written informed consent was obtained from all patients or their legal guardians when <18 years of age.

The patients were grouped according to the European Association for the Study of the Liver 2017 Clinical Practice Guidelines on managing HBV infection [3] (Table S1). Table S2 shows the numbers of patients in the HBV-DNA (++) chronic HBV-DNA infection group (group 1), HBV-DNA (++) chronic hepatitis group (group 2), HBV-DNA (–) chronic HBV infection (CHB) group (group 3), HBV-DNA (+) chronic hepatitis group (group 4), HBV-DNA (–) group (group 5), and normal group (group 6).


**Informed consent:** Informed consent has been obtained from all individuals included in this study.
**Ethical approval:** The research related to human use has been complied with all the relevant national regulations, institutional policies and in accordance with the tenets of the Helsinki Declaration, and has been approved by the North Sichuan Medical College-2020 NSMC, number 10.

### 2.2 Exosomes isolation

The exosomes were isolated from the serum samples using the ExoQuick-TC exosome extraction kit [15]. For identification, the exosomes were resuspended in 1× phosphate-buffered saline and examined by electron microscopy. The expression of ALIX, CD9, and CD63 was detected by western blot (#FD006, Biyuntian Bio) [16].

### 2.3 Nanoparticle tracking analysis (NTA)

A particle size analyzer (Particle Metrix GmbH, Ammersee, Germany) was used for NTA to determine the size and number of serum exosomes.

### 2.4 Cell culture

HepG2 cells (Saibo Kang Biotechnology Co., Shanghai, China) were cultured in DMEM with 10% fetal bovine serum without exosomes. The exosomes were fixed using 1 ml of 4% paraformaldehyde and stained with DIO (Dioc18(3) (# 40725ES10, YEASEN) at 2 mM in DMSO and diluted before use to 2 µM) for 20 min at 37°C. The exosomes were centrifuged at 1,000 rpm for 5 min, and the supernatant was removed. The exosomes were resuspended in DMEM and transferred to the HepG2 cells. After 24 h, the cell morphology was observed under fluorescence microscopy.

### 2.5 Extraction of HBV-DNA

First, 400 μl of HBV core particle DNA extract was added to the exosomes and incubated for 15 min at 37°C. The mixture was centrifuged for 3 min at 15,000×*g*. Next, 4 μl of MgCl_2_ and RNase and 5 μl of DNase I was added and incubated for 3–4 h at 37°C. Then, 200 μl of PEG8000 was added, mixed well, and incubated on ice for 40 min, followed by centrifugation at 12,000×*g*, 4°C for 7 min. The supernatant was discarded. Then, 400 μl of digestion solution was added, mixed, and added with 15 μl of proteinase K. After overnight digestion at 45°C, 400 μl of phenol chloroform isoamyl alcohol mixture (25:24:1) was added. The mixture was centrifuged at 14,000×*g* for 3 min. An equal volume of isopropanol was mixed with the supernatant, mixed, and centrifuged for 11 min at 16,500×*g* and 4°C. The supernatant was discarded, the pellet was washed with 70% ethanol, and resuspended in ddH_2_O after drying.

### 2.6 Quantitative polymerase chain reaction (qPCR)

An SYBR kit (# CW0957m, CWBIO) was used for the fluorescence quantification of the exosomes. The primers for HBV-DNA were forward 5′-ACC GAC CTT GAG GCA TAC TT-3′ and reverse 5′-GCC TAC AGC CTC CTA GTA CA-3′. The reaction system included 12.5 μl of 2× UltraSYBR Mixture, 1 μl of each HBV-DNA primer 10 μM, 8.5 μl of ddH_2_O, and 2 μl of 1/100 diluted template DNA. The thermal cycling conditions were (1) 95°C for 10 min as an initial denaturation step and (2) 39 cycles of 95°C for 15 s and 60°C for 1 min. The absolute quantification of HBV-DNA was generated based on the Ct value with the set dissociation curve.

### 2.7 Statistical analysis

SPSS 23.0 (IBM) was used for statistical analysis. Continuous variables were presented as means ± standard error (serum and exosomal HBV-DNA levels) or means ± standard deviation (other continuous variables) and compared with one-way analysis of variance; as the variances of the groups were not uniform, the Games-Howell method was used for pair-wise comparison among the six groups. In each group, exosomal HBV-DNA levels were compared with the serum HBV-DNA values using a non-parametric test. The correlations between exosomal HBV-DNA and the other clinical indexes were analyzed using Spearman’s correlation. Two-sided *P*-values <0.05 were considered statistically significant.

## 3 Results

### 3.1 Clinical characteristics of the study population

Patient grouping and characteristics are shown in Table S2. The groups are presented in Table S1.

### 3.2 Identification of serum exosomes

**Figure 1:**
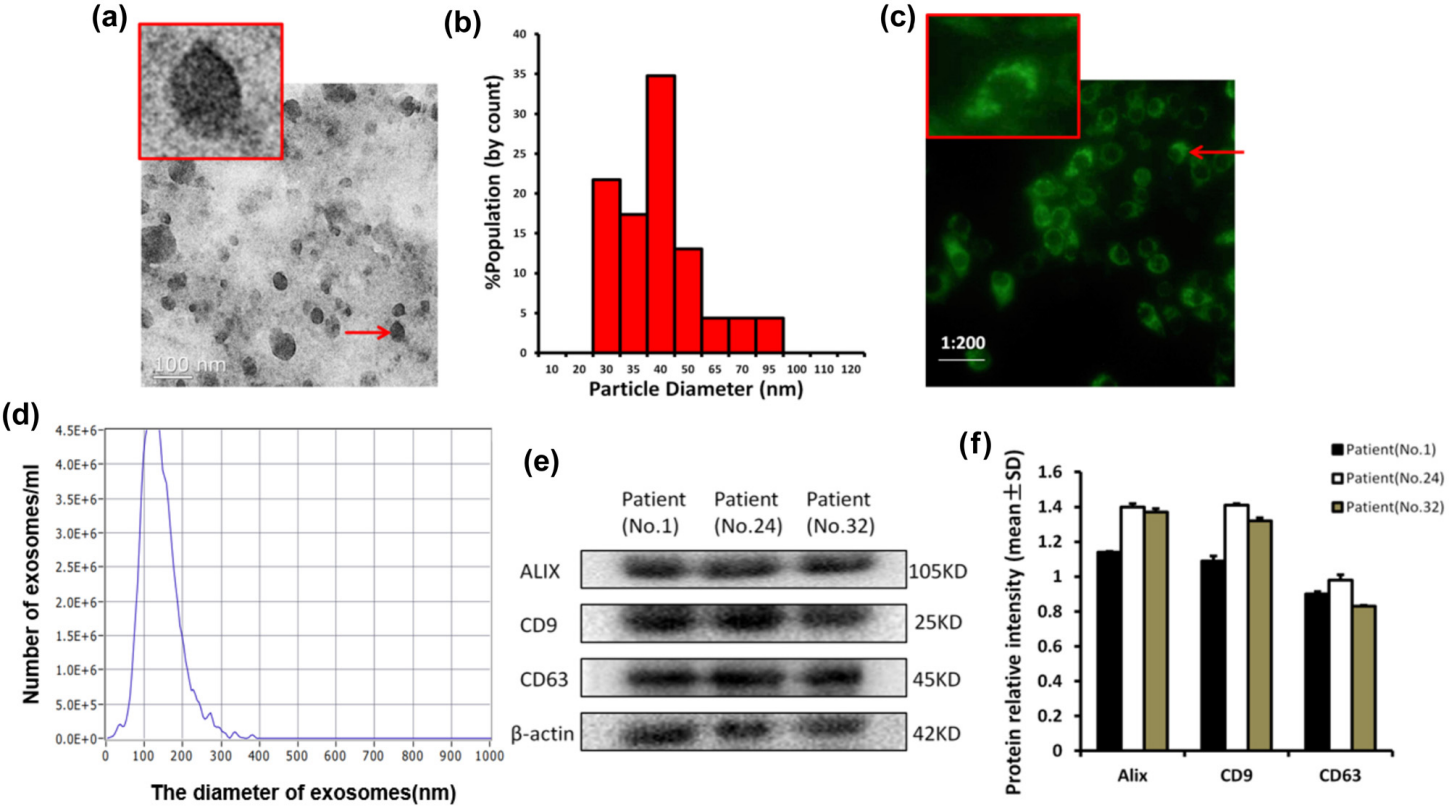
Identification of the exosomes in the serum. (a and b) Under electron microscopy, the exosomes in the serum showed a vesicular saucer-like structure with a size of about 30–95 nm. (c) After transfection of the serum exosomes into HepG2 cells for 24 h, granular fluorescence was observed in the cytoplasm. (d) Nanoparticle tracking analysis (NTA) of the nanoparticles. (e) The expression of exosome surface protein was detected by western blotting. β-Actin was used as the loading control. (f) Relative intensity of protein marker expression on the surface of exosomes in three patients.

Under electron microscopy, the serum exosomes showed an obvious saucer-like structure (Figure 1a). The diameter of the exosomes was 30–95 nm (Figure 1b). After 24 h of co-culture of the exosomes with HepG2 cells for 24 h, the cell morphology was observed using DIO staining and fluorescence microscopy, and green fluorescent exosomes were seen in the cytoplasm (Figure 1c), indicating the capacity of the exosomes to be internalized by the HepG2 cells. According to the NTA, the exosome diameter peaked at 100–125 nm (Figure 1d). The expression of Alix, CD9, and CD63 was detected using western blotting (Figure 1e and f). Therefore, the isolated particles were considered exosomes since they possessed the morphological and protein characteristics of exosomes and could be internalized by cells.

### 3.3 Expression of HBV-DNA in exosomes among the six groups

**Figure 2:**
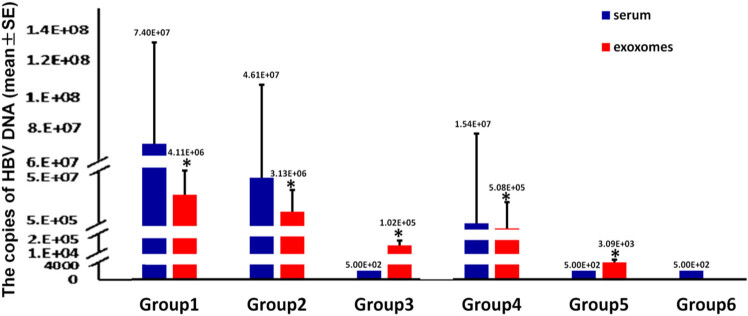
The expression of HBV-DNA in the exosomes of the six groups of serum samples. The absolute copy number of HBV-DNA in the exosomes in the six groups of serum samples detected by qPCR. *P < 0.05.

The HBV-DNA copy numbers were (4.11 ± 1.17) × 10^6^ IU/ml in group 1, (3.13 ± 0.998) × 10^6^ IU/ml in group 2, (1.02 ± 0.149) × 10^5^ IU/ml in group 3, (5.08 ± 2.65) × 10^5^ IU/ml in group 4, (3.10 ± 0.478) × 10^3^ IU/ml in group 5, and 0 IU/ml in group 6. There were significant differences between each pair of groups (all *P* < 0.05), except for group 1 vs. group 2 and group 3 vs. group 4. The HBV-DNA content was lower in the exosomes than in the serum for groups 1, 2, and 4 but higher in the exosomes in groups 3 and 5 (all *P* < 0.05) (Figure 2). Therefore, HBV-DNA can be detected in serum exosomes, and the HBV-DNA levels vary among the different groups of patients.

### 3.4 Exosomal HBV-DNA in groups with negative serum HBV-DNA

**Figure 3:**
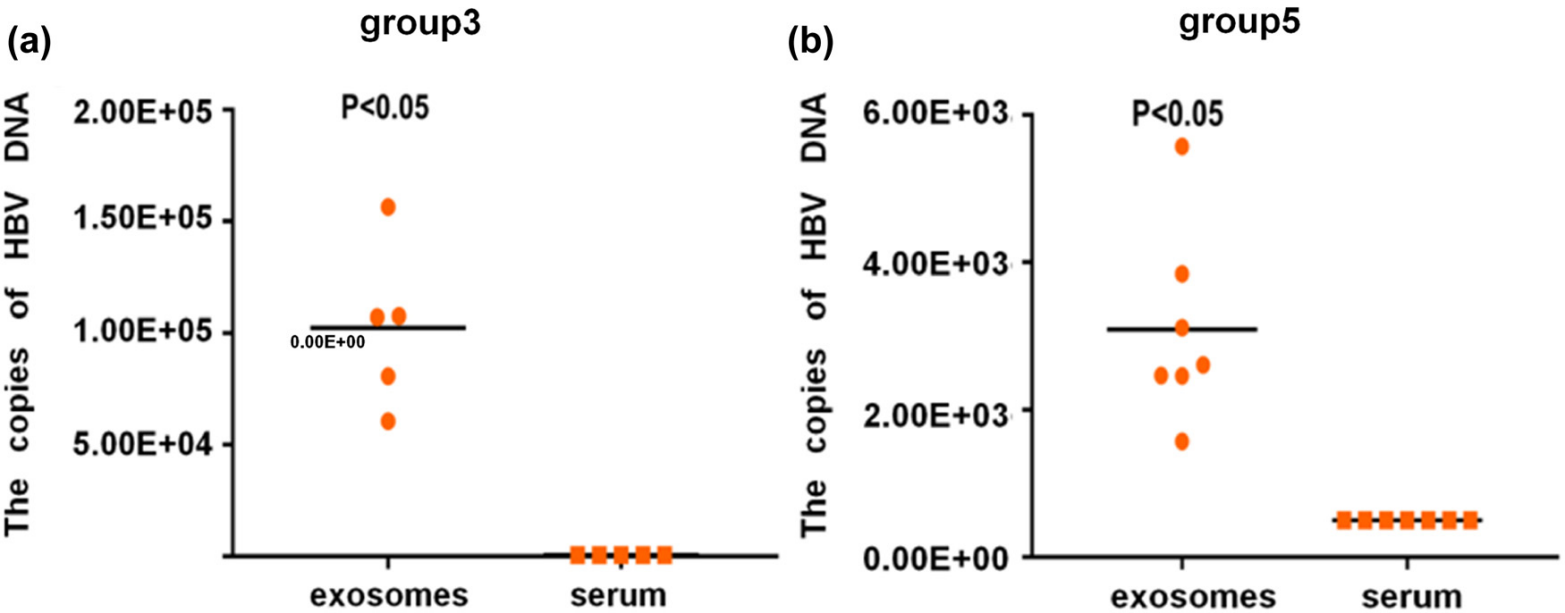
Comparison of HBV-DNA between serum and exosomes in patients with negative serum HBV-DNA. (a) The copies of HBV-DNA in serum and exosomes in group 3 patients. (b) The copies of HBV-DNA in serum and exosomes in group 5 patients. *P < 0.05.

Since exosomal HBV-DNA has a potential diagnostic value, the exosomal HBV-DNA levels were examined in patients with negative serum HBV-DNA, i.e., groups 3 (HBV-DNA-negative HBeAb-positive, normal ALT) and 5 (HBV-DNA-negative, HBcAb-positive). In groups 3 and 5, the exosomal HBV-DNA levels were higher than the serum HBV-DNA levels (all *P* < 0.05) (Figure 3). Figure S1 presents the comparisons in each patient. In group 3, the exosomal HBV-DNA levels were from 6.06 × 10^4^ to 1.56 + 05 IU/ml, while the serum HBV-DNA levels were below the detection threshold of 5.00 × 10^2^ IU/ml. In group 5, exosomal HBV-DNA levels were from 1.57 × 10^3^ to 5.57U + 03 IU/ml, while serum HBV-DNA levels were also below the detection threshold of 5.00 × 10^2^ IU/ml. In groups 3 and 5, the average copy numbers of HBV-DNA in exosomes were 1.02 × 10^5^ IU/ml (i.e., about 200 times the lowest serum HBV-DNA detection limit (500 IU/ml)) and 3.10 × 10^3^ IU/ml (i.e., about 6 times the lowest serum HBV-DNA detection limit (500 IU/ml)), respectively, showing that the former was exosomes and the latter was serum (Figure 3). These results indicate that exosomal HBV-DNA could be used to monitor treatment effects in CHB patients with negative serum HBV-DNA.

### 3.5 Exosomal HBV-DNA in groups with positive serum HBV-DNA

**Figure 4:**
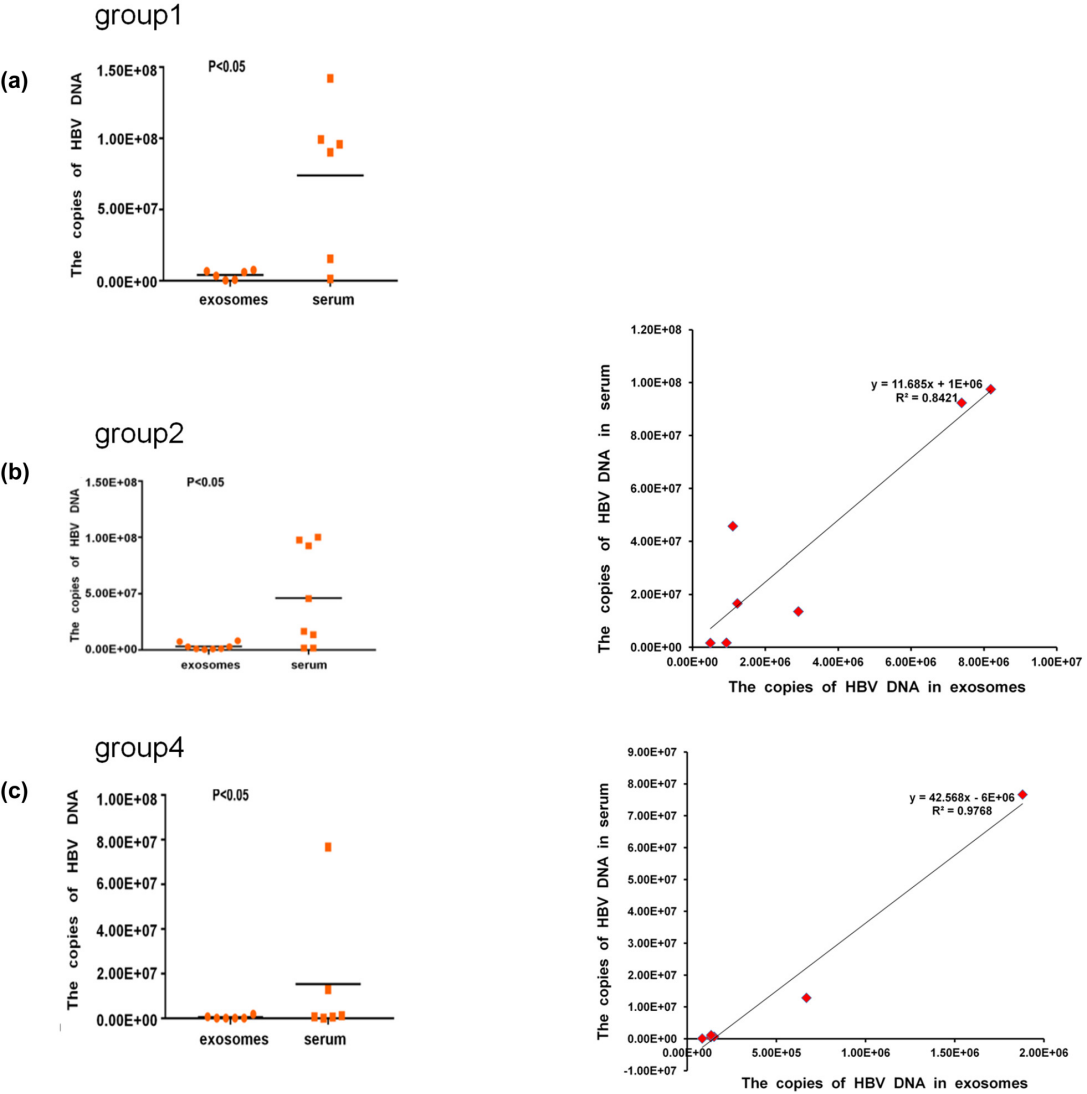
Comparison and correlation (Pearson’s) of HBV-DNA between serum and exosomes in patients with positive serum HBV-DNA. (a) Group 1. (b) Group 2. (c) Group 4. *P < 0.05.

In the HBV-DNA (+) group, only the exosomal HBV-DNA in groups 2 and 4 were correlated with the serum HBV-DNA, while there were no correlations in group 1 (because the ALT/AST levels are normal, indicating less cell damage; the release of exosomes was smaller, and the detected HBV in the serum exosomes was not related to HBV in serum). Figure 4 shows the comparison of exosomal and serum HBV-DNA in patients with positive serum HBV-DNA (groups 1, 2, and 4). The serum HBV-DNA levels were higher than the exosomal HBV-DNA levels (all *P* < 0.05). Figure S1 presents the comparisons in each patient. All patients had higher serum HBV-DNA levels than the exosomal HBV-DNA. Still, some patients presented high exosomal HBV-DNA levels (patients 13, 14, 20, 21, and 25). ALT and AST levels indicate the degree of liver damage. Exosomes are derived from cells. Damaged and ruptured cells can release more exosomes. If the liver cells are not damaged, the release of exosomes should be limited, resulting in that HBV in the liver would not be related to the serum levels. The exosomal and serum HBV-DNA levels were correlated in groups 2 (*R* ^2^ = 0.84) and 4 (*R* ^2^ = 0.98). The results indicate that as serum HBV-DNA, exosomal HBV-DNA can represent the disease activity in patients with positive serum HBV-DNA. When the serum HBV-DNA is high (groups 1 and 2; HBV-DNA > 2 × 10^5^ IU/ml), the serum has a larger volume to accommodate HBV-DNA than exosomes, and thus, more HBV-DNA is detected in the serum. Serum HBV-DNA in group 1 was 4.35–27.45 times that of exosomal HBV-DNA, while serum HBV-DNA in group 2 was 1.84–41.17 times that of exosomal HBV-DNA. When serum HBV-DNA levels were moderate, such as in group 4 (HBV-DNA > 2 × 10^3^ IU/ml), the serum HBV-DNA levels were still higher than that of exosomes, and the difference was 1.73–40.80 times (*P* < 0.05). When serum HBV-DNA was negative, such as in groups 3 and 5 (HBV-DNA < 5 × 10^2^ IU/ml), there was still virus replication even if the HBV-DNA in serum was undetectable, but exosomal HBV-DNA was detectable because of their key function in replication.

### 3.6 Correlations of exosomal HBV-DNA with liver function markers

**Figure 5:**
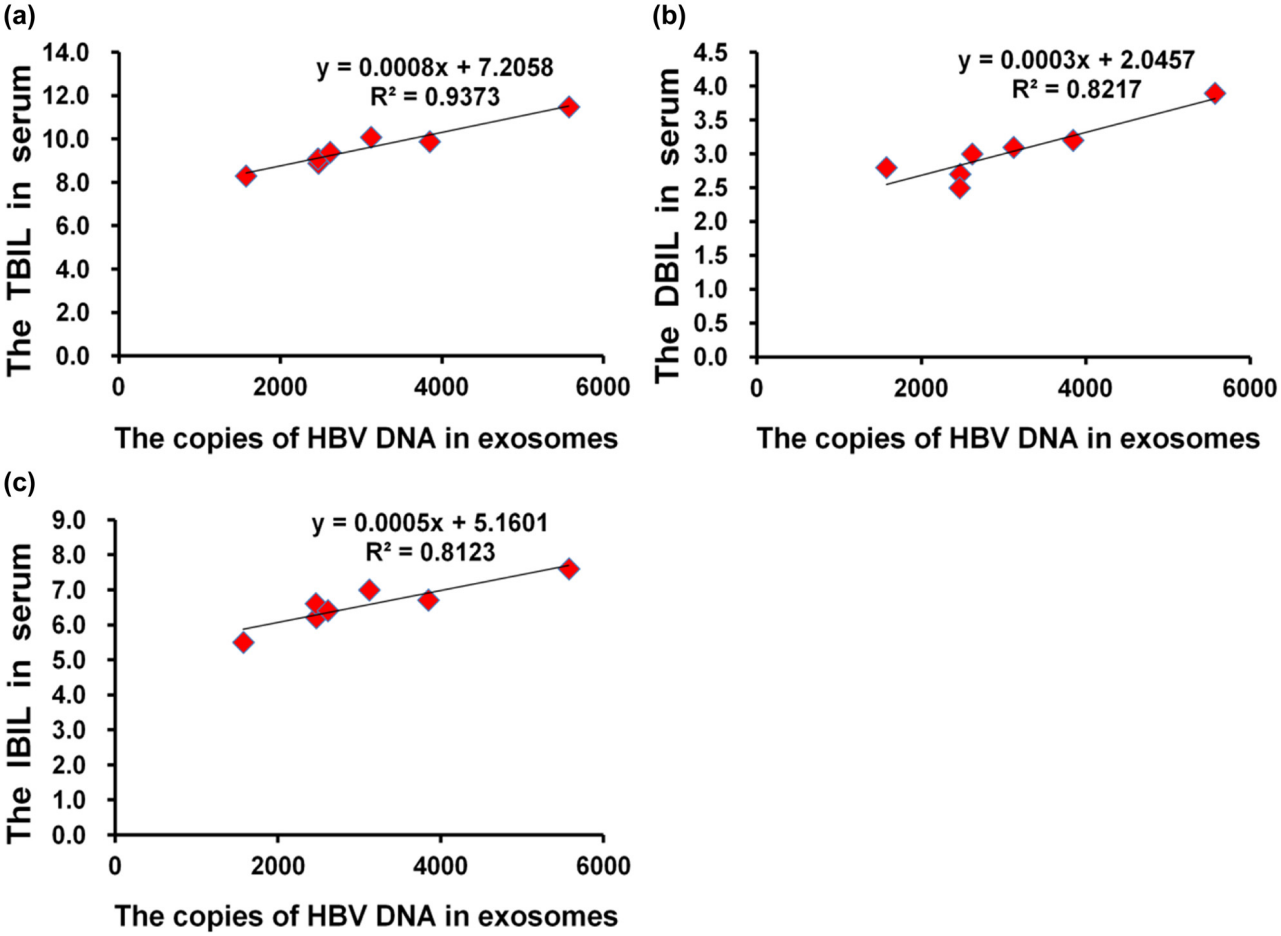
Correlations of exosomal HBV-DNA with liver function markers. (a) Total bilirubin (TBIL). (b) Direct bilirubin (DBIL). (c) Indirect bilirubin (IBIL).

Since exosomal HBV-DNA could be clinically used in serum HBV-DNA-negative patients, the exosomal HBV-DNA levels were examined for correlation with liver function markers. The exosomal HBV-DNA levels were correlated with TBIL (*R* ^2^ = 0.94) (Figure 5a), DBIL (*R* ^2^ = 0.82) (Figure 5b), and IBIL (*R* ^2^ = 0.81) (Figure 5c) in group 5 (all *P* < 0.05). In the other groups, there were no correlations between exosomal HBV-DNA and liver function markers (Tables S3–S6; all *P* > 0.05). Therefore, the results support the clinical usefulness of exosomal HBV-DNA levels for monitoring CHB patients with negative serum HBV-DNA.

## 4 Discussion

Serum HBV-DNA and ALT are routinely used as key clinical indicators to diagnose and evaluate patients with CHB [17,18]. The best opportunity for antiviral therapy is the presence of serum HBV-DNA and ALT elevation [3,19]. Still, some patients have low-elevated HBV-DNA and normal ALT, and it is difficult to determine the treatment timing for such patients [19,20]. These indicators might not completely reflect the severity of the liver lesions, and treatment indication then depends on liver biopsy [21,22,23].

Exosomes play crucial roles in communication among cells and might be diagnostic and prognostic indicators for various diseases [23,24,25,26]. This study indicates that the exosome copy numbers of HBV-DNA (1.02 × 10^5^ and 3.10 × 10^3^ IU/ml) were far higher than the detection limit of serum HBV-DNA (5 × 10^2^ IU/ml) in groups 3 and 5, suggesting that exosomal HBV-DNA might be more sensitive than serum HBV-DNA. Exosomal HBV-DNA is more sensitive than serum HBV-DNA when serum HBV-DNA is negative. Interestingly, for patients whose serum HBV-DNA levels indicated a moderate-high replication level (>2 × 10^3^ IU/ml) (group 4), the copy numbers of exosomal HBV-DNA (1.02 × 10^5^ IU/ml) (group 3) were comparable to the exosomal HBV-DNA levels (5.08 × 10^5^ IU/ml) (group 4) from the HBV-DNA-positive patients (*P* > 0.05). It suggests that the serum HBV-DNA levels might not reflect the true status of HBV replication in disease progression.

When serum HBV-DNA levels were high, there was no correlation between exosomal HBV-DNA and liver function-related indicators in group 1, while there were correlations in groups 2 and 4, which can be due to liver damage. On the other hand, when serum HBV-DNA is low, exosomal HBV-DNA has a certain correlation with liver function-related indicators, such as in the HBV-DNA-negative group (group 5). This study showed that exosomal HBV-DNA was positively correlated with TBIL, DBIL, and IBIL, indicating that the exosomal HBV-DNA levels could reflect liver injury to a certain extent. It should also be noted that ALT, an important biochemical indicator of liver injury, showed no correlation to the HBV-DNA levels in serum and exosomes.

Combined with clinical diagnosis and treatment standards, when HBV-DNA is >2 × 10^5^ IU/ml and ALT is normal, there is a controversy about the use of antiviral treatment [3]. Comprehensive judgment is needed to fully consider patients’ age, liver histological examination, and liver compensation since such patients lack simple indicators to reflect the timing of treatment accurately. For example, in group 1, serum HBV-DNA was high, while ALT was normal, and such patients could not be treated according to clinical treatment guidelines. Still, this study showed that the exosomal HBV-DNA copy number in group 1 was 4.11 × 10^6^ IU/ml, i.e., 1.3 times higher than the exosomal HBV-DNA copy number in group 2. On the other hand, eight patients (HBeAg (+), serum HBV-DNA >2 × 10^3^ IU/ml, and ALT > ULN) in group 2 absolutely met the guideline criteria for antiviral treatment, but their exosomal HBV-DNA levels were only 1.10 × 10^6^ IU/ml, far lower than that of group 1. Based on this inference, antiviral therapy may be given to patients in group 1 when exosomal HBV-DNA is >3.13 × 10^5^ IU/ml. Meanwhile, the patients in groups 3 and 5 did not meet the indications for clinical antiviral therapy. The average copy numbers of HBV-DNA in the exosomes and serum were 1.02 × 10^5^ and 3.10 × 10^3^ IU/ml, respectively, about 6–200 times higher in exosomes than in serum. Therefore, the lower limit of exosomal HBV-DNA could be >1.56 × 10^5^ IU/ml for antiviral therapy. Exosomes could be used as a simple and non-invasive detection indicator for monitoring CHB [27,28,29].

Recent studies showed that exosomes could regulate the immune response during HBV infection. Immune escape by down-regulating IL-12 to inhibit the activity of NK cells is one of the important reasons for persistent virus infection [30,31]. The insidious repeated activities of the virus can cause severe liver disease. During the disease, there is a certain risk in determining the timing of treatment based on the serum HBV-DNA, ALT, and other indicators specified in the guidelines. For example, in patients with negative HBV-DNA, relying on the existing indicators (serum HBV-DNA, ALT, BIL, etc.) is not enough to determine the timing of treatment correctly. At this time, more sensitive, accurate, and stable indicators are needed to reflect the levels of HBV-DNA to determine treatment. This study also reveals that exosomes can play an important role in monitoring CHB. The possible reason is that HBV-DNA enters exosomes through a ceramide-dependent pathway [32]. The exosomes act as an important vector to transport HBV-DNA into the liver cells to promote the massive replication of HBV in the body [33,34]. Exosomes are not easily diluted by the body’s blood and remain stable in body fluids. Therefore, monitoring the level of HBV-DNA in exosomes can timely and accurately reflect the occurrence and progress of the disease and determine the treatment to prevent the disease from turning into liver cirrhosis and cancer.

## 5 Conclusion

This study suggests that when a patient’s serum HBV-DNA is negative, exosomal HBV-DNA can accurately reflect the level of HBV replication *in vivo*. In serum HBV-DNA-negative patients, exosomal HBV-DNA were strongly and positively correlated with TBIL, DBIL, and IBIL.

## Supplementary Material

Supplementary material

## References

[1] Jefferies M, Rauff B, Rashid H, Lam T, Rafiq S. Update on global epidemiology of viral hepatitis and preventive strategies. World J Clin Cases. 2018;6(13):589–99. 10.12998/wjcc.v6.i13.589 PMC6232563 30430114

[2] Terrault NA, Lok ASF, McMahon BJ, Chang KM, Hwang JP, Jonas MM, et al. Update on prevention, diagnosis, and treatment of chronic hepatitis B: AASLD 2018 hepatitis B guidance. Hepatology. 2018;67(4):1560–99. 10.1002/hep.29800 PMC5975958 29405329

[3] Lampertico P, Agarwal K, Berg T, Buti M, Janssen HL, Papatheodoridis G, et al. EASL 2017 Clinical Practice Guidelines on the management of hepatitis B virus infection. J Hepatol. 2017;67(2):370–98. 10.1016/j.jhep.2017.03.021 28427875

[4] Mathieu M, Martin-Jaular L, Lavieu G, Thery C. Specificities of secretion and uptake of exosomes and other extracellular vesicles for cell-to-cell communication. Nat Cell Biol. 2019;21(1):9–17. 10.1038/s41556-018-0250-9 30602770

[5] Hessvik NP, Llorente A. Current knowledge on exosome biogenesis and release. Cell Mol Life Sci. 2018;75(2):193–208. 10.1007/s00018-017-2595-9 PMC5756260 28733901

[6] Sun Y, Liu J. Potential of cancer cell-derived exosomes in clinical application: a review of recent research advances. Clin Ther. 2014;36(6):863–72. 10.1016/j.clinthera.2014.04.018 24863262

[7] Caby MP, Lankar D, Vincendeau-Scherrer C, Raposo G, Bonnerot C. Exosomal-like vesicles are present in human blood plasma. Int Immunol. 2005;17(7):879–87. 10.1093/intimm/dxh267 15908444

[8] Fang C, Wei Y. Research status of extracellular vesicles in liver disease. Med Rev. 2017;23(16):3142–45.

[9] Crenshaw BJ, Gu L, Sims B, Matthews QL. Exosome biogenesis and biological function in response to viral infections. Open Virol J. 2018;12:134–48. 10.2174/1874357901812010134 PMC6187740 30416610

[10] Kapoor NR, Chadha R, Kumar S, Choedon T, Reddy VS, Kumar V. The HBx gene of hepatitis B virus can influence hepatic microenvironment via exosomes by transferring its mRNA and protein. Virus Res. 2017;240:166–74. 10.1016/j.virusres.2017.08.009 28847700

[11] Tian XP, Wang CY, Jin XH, Li M, Wang FW, Huang WJ, et al. Acidic microenvironment up-regulates exosomal miR-21 and miR-10b in early-stage hepatocellular carcinoma to promote cancer cell proliferation and metastasis. Theranostics. 2019;9(7):1965–79. 10.7150/thno.30958 PMC6485281 31037150

[12] Wang H, Hou L, Li A, Duan Y, Gao H, Song X. Expression of serum exosomal microRNA-21 in human hepatocellular carcinoma. Biomed Res Int. 2014;2014:864894. 10.1155/2014/864894 PMC4052145 24963487

[13] De Toro J, Herschlik L, Waldner C, Mongini C. Emerging roles of exosomes in normal and pathological conditions: new insights for diagnosis and therapeutic applications. Front Immunol. 2015;6:203. 10.3389/fimmu.2015.00203 PMC4418172 25999947

[14] Urbanelli L, Buratta S, Tancini B, Sagini K, Delo F, Porcellati S, et al. The role of extracellular vesicles in viral infection and transmission. Vaccines (Basel). 2019;7(3):102. 10.3390/vaccines7030102 PMC6789493 31466253

[15] Cheng Y, Qu X, Dong Z, Zeng Q, Ma X, Jia Y, et al. Comparison of serum exosome isolation methods on co-precipitated free microRNAs. PeerJ. 2020;8:e9434. 10.7717/peerj.9434 PMC7457927 32923177

[16] Simons M, Raposo G. Exosomes–vesicular carriers for intercellular communication. Curr Opin Cell Biol. 2009;21(4):575–81. 10.1016/j.ceb.2009.03.007 19442504

[17] Terrault NA, Bzowej NH, Chang KM, Hwang JP, Jonas MM, Murad MH, et al. AASLD guidelines for treatment of chronic hepatitis B. Hepatology. 2016;63(1):261–83. 10.1002/hep.28156 PMC5987259 26566064

[18] Katwaroe WK, Brakenhoff SM, van der Spek DPC, de Knegt RJ, van Kleef LA, de Man RA, et al. Assessment of adherence to clinical guidelines in patients with chronic hepatitis B. Viruses. 2022;14(10):2229. 10.3390/v14102229 PMC9607053 36298784

[19] Wong GL. Management of chronic hepatitis B patients in immunetolerant phase: what latest guidelines recommend. Clin Mol Hepatol. 2018;24(2):108–13. 10.3350/cmh.2017.0068 PMC6038942 29353469

[20] Ren S, Wang W, Lu J, Wang K, Ma L, Zheng Y, et al. Effect of the change in antiviral therapy indication on identifying significant liver injury among chronic hepatitis B virus infections in the grey zone. Front Immunol. 2022;13:1035923. 10.3389/fimmu.2022.1035923 PMC9647141 36389814

[21] Wong GL. Prediction of fibrosis progression in chronic viral hepatitis. Clin Mol Hepatol. 2014;20(3):228–36. 10.3350/cmh.2014.20.3.228 PMC4197170 25320725

[22] Chinese Medical Association. Expert opinion on expanding anti-HBV treatment for chronic hepatitis B. Zhonghua Gan Zang Bing Za Zhi. 2022;30(2):131–36. 10.3760/cma.j.cn501113-20220209-00060 PMC12770186 35359064

[23] Wang F, Li L, Piontek K, Sakaguchi M, Selaru FM. Exosome miR-335 as a novel therapeutic strategy in hepatocellular carcinoma. Hepatology. 2018;67(3):940–54. 10.1002/hep.29586 PMC5826829 29023935

[24] Xie JY, Wei JX, Lv LH, Han QF, Yang WB, Li GL, et al. Angiopoietin-2 induces angiogenesis via exosomes in human hepatocellular carcinoma. Cell Commun Signal. 2020;18(1):46. 10.1186/s12964-020-00535-8 PMC7077328 32183816

[25] Cho HJ, Eun JW, Baek GO, Seo CW, Ahn HR, Kim SS, et al. Serum exosomal MicroRNA, miR-10b-5p, as a potential diagnostic biomarker for early-stage hepatocellular carcinoma. J Clin Med. 2020;9(1):281. 10.3390/jcm9010281 PMC7019940 31968558

[26] Jahangiri L, Ishola T. Exosomes in neuroblastoma biology, diagnosis, and treatment. Life (Basel). 2022;12(11):1714. 10.3390/life12111714 PMC9694311 36362869

[27] Li S, Li S, Wu S, Chen L. Exosomes modulate the viral replication and host immune responses in HBV infection. Biomed Res Int. 2019;2019:2103943. 10.1155/2019/2103943 PMC6558633 31275965

[28] Ferrantelli F, Manfredi F, Chiozzini C, Anticoli S, Olivetta E, Arenaccio C, et al. DNA vectors generating engineered exosomes potential CTL vaccine candidates against AIDS, hepatitis B, and tumors. Mol Biotechnol. 2018;60(11):773–82. 10.1007/s12033-018-0114-3 30167966

[29] Ye B, Shen Y, Chen H, Lin S, Mao W, Dong Y, et al. Differential proteomic analysis of plasma-derived exosomes as diagnostic biomarkers for chronic HBV-related liver disease. Sci Rep. 2022;12(1):14428. 10.1038/s41598-022-13272-4 PMC9402575 36002595

[30] Kouwaki T, Fukushima Y, Daito T, Sanada T, Yamamoto N, Mifsud EJ, et al. Extracellular vesicles including exosomes regulate innate immune responses to hepatitis B virus infection. Front Immunol. 2016;7:335. 10.3389/fimmu.2016.00335 PMC5005343 27630638

[31] Amin OE, Colbeck EJ, Daffis S, Khan S, Ramakrishnan D, Pattabiraman D, et al. Therapeutic potential of TLR8 agonist GS-9688 (Selgantolimod) in chronic hepatitis B: remodeling of antiviral and regulatory mediators. Hepatology. 2021;74(1):55–71. 10.1002/hep.31695 PMC8436741 33368377

[32] Sanada T, Hirata Y, Naito Y, Yamamoto N, Kikkawa Y, Ishida Y, et al. Transmission of HBV DNA mediated by ceramide-triggered extracellular vesicles. Cell Mol Gastroenterol Hepatol. 2017;3(2):272–83. 10.1016/j.jcmgh.2016.10.003 PMC5331779 28275693

[33] Yang Y, Han Q, Hou Z, Zhang C, Tian Z, Zhang J. Exosomes mediate hepatitis B virus (HBV) transmission and NK-cell dysfunction. Cell Mol Immunol. 2017;14(5):465–75. 10.1038/cmi.2016.24 PMC5423088 27238466

[34] Gan W, Chen X, Wu Z, Zhu X, Liu J, Wang T, et al. The relationship between serum exosome HBV-miR-3 and current virological markers and its dynamics in chronic hepatitis B patients on antiviral treatment. Ann Transl Med. 2022;10(10):536. 10.21037/atm-22-2119 PMC9201139 35722385

