# Supplementary material for: Exosomal HBV-DNA for diagnosis and treatment monitoring of chronic hepatitis B
Source: Open Life Sci. 2023 Apr 15;18(1):20220585. doi: 10.1515/biol-2022-0585 (PMC10106972; doi:10.1515/biol-2022-0585)
Supplement: Supplementary material [file biol-2022-0585-sm.pdf]

Supplementary material

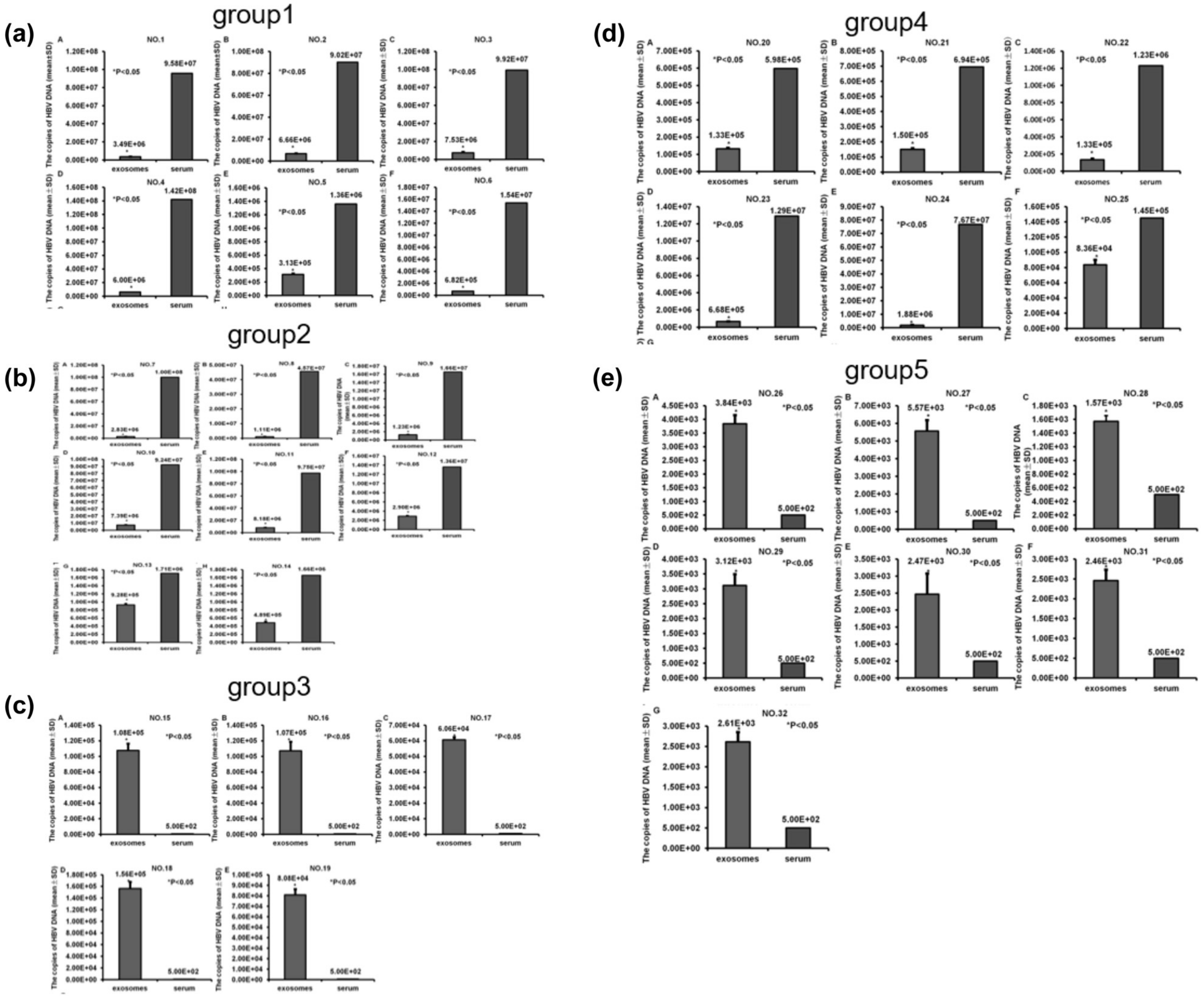

Figure S1: Comparisons of the exosomal HBV-DNA levels in each patient.

Table S1: Patient classification according to the EASL 2017 Clinical Practice Guidelines on managing HBV infection

Group	HBV-DNA titer (IU/ml)	HBV-DNA	HbeAg	ALT	HbeAb	HbsAg	HBbAb	HbcAb
1	$>2 \times 10^5$	++	+	Normal	NC	NC	NC	NC
2	NC	++	+	Elevated	NC	NC	NC	NC
3	$<5 \times 10^2$	Negative	Negative	Normal	NC	NC	NC	NC
4	$>2 \times 10^3$	+	Negative	Elevated	+	NC	NC	NC
5	NC	Negative	Negative	Normal	NC	Negative	Negative	+
6	NC	Negative	Negative	Normal	NC	Negative	Negative	Negative

NC: not considered in the group definition.

Table S2: Test results of the six groups of patients

	Sex	Age	HBV-DNA (IU/ml)	HBsAg	HBeAg	HBeAb	HBcAb	AST (U/l)	ALT (U/l)	TBIL (μmol/l)
**Group 1**										
No. 1	Female	39	9.58×10^7	>52,000	2067	11.21	0.01	30	31	8.6
No. 2	Male	25	9.02×10^7	44,635	1990	8.11	0.01	30	29	27.8
No. 3	Female	20	9.92×10^7	33,081	1771	8.58	0.01	32	25	14.3
No. 4	Male	20	1.42×10^8	39,886	2079	7.31	0.01	20	25	24.1
No. 5	Female	49	1.36×10^6	24,294	1695	5.52	0.01	55	38	6.8
No. 6	Female	46	1.54×10^7	11,738	32	1.23	0.01	40	25	10.5
**Group 2**										
No. 7	Female	17	$>1.00 \times 10^8$	33,170	1736	7.33	0.01	65	70	9.4
No. 8	Male	30	4.57×10^7	12,477	1562	6.97	0.01	73	206	9.0
No. 9	Male	34	1.66×10^7	6302	854	4.43	0.01	75	112	14.5
No. 10	Female	11	9.24×10^7	46,999	2074	10.75	0.01	47	59	7.6
No. 11	Female	21	9.75×10^7	35,726	1733	7.22	0.01	37	56	10.9
No. 12	Male	23	1.36×10^7	6468	1581	7.64	0.01	161	307	15.6
No. 13	Male	42	1.71×10^6	7280	8553	3.46	0.01	38	73	9.2
No. 14	Female	46	1.66×10^6	9887	1766	8.50	0.01	50	56	7.0
**Group 3**										
No. 15	Male	58	<500	<0.05	0.097	0.01	0.01	30	28	8.0
No. 16	Female	38	<500	<0.05	0.1	0.88	0.01	10	18	13.0
No. 17	Male	52	<500	<0.05	0.097	0.3	0.01	29	26	14.1
No. 18	Male	59	<500	<0.05	0.098	0.11	0.01	19	17	12.7
No. 19	Male	56	<500	201.8	0.095	0.041	0.01	71	23	22.0
**Group 4**										
No. 20	Male	63	5.98×10^5	26,019	0.088	0	0.01	1243	820	159.3
No. 21	Male	29	6.94×10^5	357	0.32	0.35	0.01	297	664	24.5
No. 22	Female	48	1.23×10^6	765.9	0.102	0	0.01	70	58	10.8
No. 23	Male	62	1.29×10^7	931.4	0.094	0.01	0.01	122	206	21.1
No. 24	Female	45	7.67×10^7	6573	0.088	0.01	0.01	88	97	12.4
No. 25	Female	42	1.45×10^5	1278	0.089	0	0.01	32	42	8.9
**Group 5**										
No. 26	Female	65	<500	<0.05	0.08	1.08	0.01	20	33	9.9
No. 27	Female	52	<500	<0.05	0.101	1.13	0.01	13	21	11.5
No. 28	Male	3	<500	<0.05	0.075	0.8	0.01	10	16	8.3
No. 29	Female	32	<500	<0.05	0.111	0.57	0.01	7	15	10.1
No. 30	Male	31	<500	<0.05	0.103	1.45	0.22	11	18	8.9
No. 31	Male	57	<500	<0.05	0.095	1.41	0.82	12	35	9.1
No. 32	Male	42	<500	<0.05	0.118	1.47	0.15	28	26	9.4
**Group 6**										
No. 33	Male	53	<500	<0.05	0.08	1.48	1.42	13	17	14.2
No. 34	Female	29	<500	<0.05	0.118	1.43	2.19	29	24	13.1
No. 35	Female	40	<500	<0.05	0.093	1.53	2.14	11	14	8.5

(*Continued*)

Table S2: *Continued*

	Sex	Age	HBV-DNA (IU/ml)	HBsAg	HBeAg	HBeAb	HBcAb	AST (U/l)	ALT (U/l)	TBIL (μmol/l)
No. 36	Female	52	<500	<0.05	0.091	1.44	1.93	21	20	11.3
No. 37	Female	23	<500	<0.05	0.103	1.37	2.31	14	21	13.5
No. 38	Male	7	<500	<0.05	0.106	1.4	1.63	39	22	14.5
No. 39	Female	22	<500	<0.05	0.122	1.5	2.15	9	18	10.5

Table S3: Correlation between the expression of HBV-DNA in exosomes of chronic HBV infection group (HBV-DNA (++), and normal ALT) and the clinical indicators

Variable	*P*	*r*
HBV-DNA (IU/ml)	0.111	0.714
HBsAg	0.285	0.6
HBeAg	0.397	0.429
HBeAb	0.208	0.6
HBcAb	/	/
ALT (U/l)	0.295	−0.516
AST (U/l)	0.257	−0.551
TBIL (μmol/l)	0.072	0.771
DBIL (μmol/l)	0.072	0.771
IBIL (μmol/l)	0.072	0.771

Table S5: Correlation between the expression of HBV-DNA in exosomes of chronic HBV infection group (HBV-DNA (−) and normal ALT) and the clinical indicators

Variable	*P*	*r*
HBV-DNA (IU/ml)	/	/
HBsAg	/	/
HBeAg	0.434	0.462
HBeAb	0.624	−0.3
HBcAb	/	/
ALT (U/l)	0.505	−0.4
AST (U/l)	0.624	−0.3
TBIL (μmol/l)	0.104	−0.8
DBIL (μmol/l)	0.104	−0.8
IBIL (μmol/l)	0.747	−0.2

Table S4: Correlation between the expression of HBV-DNA in exosomes of chronic hepatitis group (HBV-DNA (++) and elevated ALT) and the clinical indicators

Variable	*P*	*r*
HBV-DNA (IU/ml)	0.014	0.857
HBsAg	0.207	0.5
HBeAg	0.736	−0.143
HBeAb	0.456	0.31
HBcAb	/	/
ALT (U/l)	0.799	−0.108
AST (U/l)	0.736	−0.143
TBIL (μmol/l)	0.26	0.452
DBIL (μmol/l)	0.071	0.667
IBIL (μmol/l)	0.61	0.214

Table S6: Correlation between the expression of HBV-DNA in exosomes of chronic hepatitis group (HBV-DNA (+) and elevated ALT) and the clinical indicators

Variable	*P*	*r*
HBV-DNA (IU/ml)	0.005	0.943
HBsAg	0.787	−0.143
HBeAg	0.957	0.029
HBeAb	0.092	0.741
HBcAb	/	/
ALT (U/l)	0.704	0.2
AST (U/l)	0.704	0.2
TBIL (μmol/l)	0.704	0.2
DBIL (μmol/l)	0.787	0.143
IBIL (μmol/l)	0.704	0.2

Table S7: Correlation between the expression of HBV-DNA in exosomes of HBV-DNA (−) group and the clinical indicators

**Variable**	***P***	***r***
HBV-DNA (IU/ml)	/	/
HBsAg	/	/
HBeAg	0.535	0.286
HBeAb	0.819	−0.107
HBcAb	0.307	−0.453
ALT (U/l)	0.879	0.071
AST (U/l)	0.383	0.393
TBIL (μmol/l)	0.003	0.929
DBIL (μmol/l)	0.007	0.893
IBIL (μmol/l)	0.014	0.857

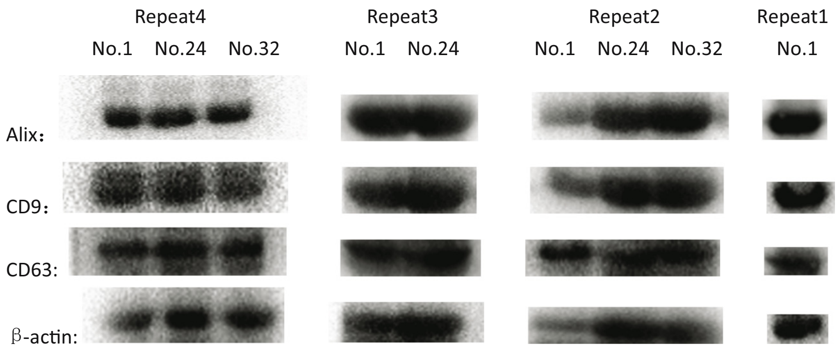

Original, uncropped and unadjusted blots Required As per Open Life Science policy, provide the original, uncropped and unadjusted blots from three experiments for review.
